# Pure sensory syndromes and post-stroke pain secondary to bilateral thalamic lacunar infarcts: a case report

**DOI:** 10.1186/1752-1947-6-359

**Published:** 2012-10-24

**Authors:** Karl B Alstadhaug, Jan F Prytz

**Affiliations:** 1 Department of Neurology, Nordland Hospital Trust, Bodø 8092, Norway; 2 Department of Radiology, Nordland Hospital Trust, Nordland, Norway; 3 Institute of Clinical Medicine, University of Tromsø, Tromsø, Norway

**Keywords:** Sensory stroke, Thalamus, Lacunar infarct, Post-stroke pain, Déjerine-Roussy syndrome

## Abstract

**Introduction:**

Patients often complain about sensory symptoms that appear to the doctor as harmless, and reassurances are often given. Sensory strokes may easily be ignored.

**Case presentation:**

A 48-year-old Caucasian woman with insulin-dependent diabetes and hyperlipidemia experienced symptoms that progressed within hours to a complete left-sided hemisensory syndrome. This was caused by a lacunar infarct in the ventral posterior tier nuclei of the right thalamus. A few days later she gradually developed an almost identical, but incomplete hemisensory syndrome on the opposite side caused by a corresponding lacune in the left thalamus. Severe persistent and paroxysmal pain on both sides of the body became disabling.

**Conclusion:**

Small strokes only affecting the somatosensory system should not be underestimated. Neuropathic pain may result. Probably unique in the present case is the demonstration of bilateral thalamic pain secondary to two almost identical thalamic infarcts. Small vessel disease (microatheroma or lipohyalinosis) was the most likely cause of the lacunes. One can only speculate if there was an occlusion in two separate thalamic perforators, or in a single dominant artery supplying the bilateral thalami.

## Introduction

Pure sensory stroke, initially described by Fisher almost 50 years ago [1], especially when tract-specific, may easily be explained away as a functional disorder. The strokes may be very small, located anywhere in the sensory pathway from the pons to the parietal cortex [2,3]. Pure sensory strokes are characterized by subjective symptoms with no evidence of weakness, speech difficulties, or other classical symptoms of cerebral infarction or hemorrhage. Symptoms may be subtle and progress within hours, may include positive phenomena like tingling, and may thus lack the characteristic apoplectic loss of function. Improvement and normalization within weeks is considered to be the rule [3]. However, central pain, which usually involves damage to the spinothalamic tract or the thalamic ventroposterior nucleus (VPN) [4,5], has been known for more than a century [6]. Here we describe a unique case with a vascular lesion in the posterolateral part of the thalamus on both sides, developing both an incomplete and a complete sensory hemisyndrome with post-stroke pain accompaniment.

## Case presentation

A 48-year-old Caucasian woman with a well-treated hypercholesterolemia and a 20-year history of adult-onset type 1 diabetes experienced over 8 hours a progressive alteration in sensations from the left side of her body (including the head), causing her to feel as if she was split in two. She reported unpleasant symptoms such as numbness, coldness and paresthesia, but was told by the doctor on call not to worry.

**Figure 1:**
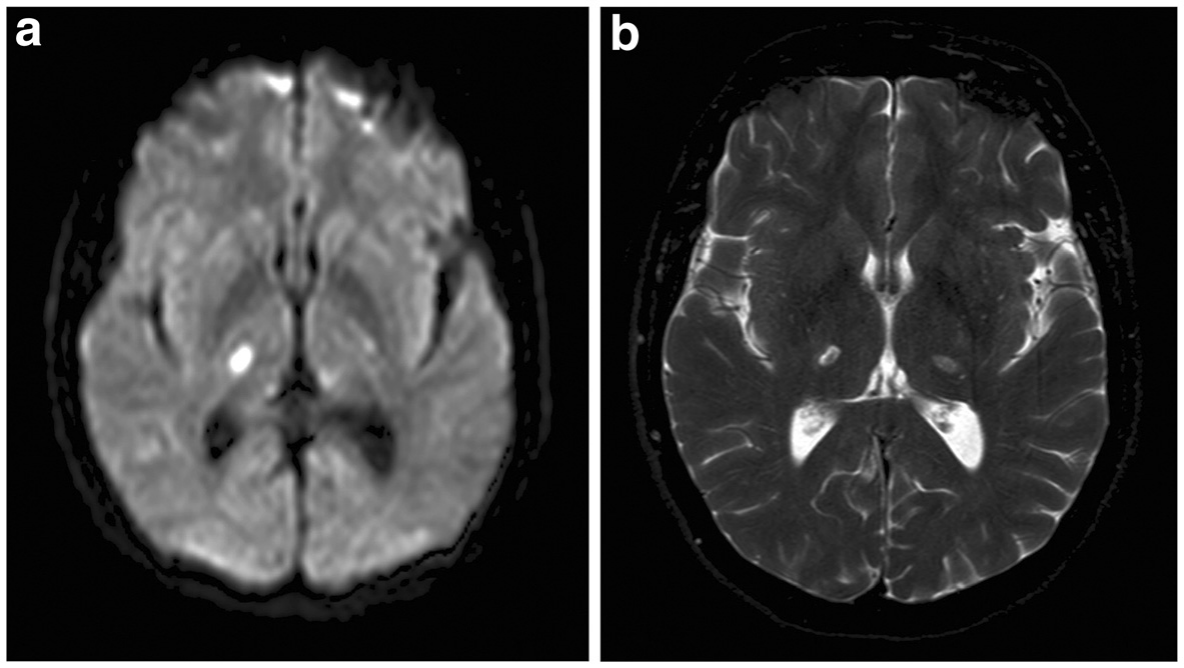
**(a) Diffusion-weighted magnetic resonance imaging 6 days after an acute infarction in the right inferolateral artery territory. **(**b**) T2-weighted magnetic resonance image a fortnight later shows bilateral infarctions.

Eventually, on examination 6 days later, the patient’s blood pressure was found to be 117/69mmHg and her pulse was 65 beats per minute and regular. A slight dysesthesia was reported on touch and pinprick in a complete hemisensory pattern. Her joint position, temperature and vibration sensation were preserved. However, a slight ataxia in her left leg was demonstrated. Magnetic resonance imaging (MRI) revealed a lacunar infarct in her right thalamus (Figure 1a). Except for glycated hemoglobin (HbA_1c_) above 10%, a thorough examination with regard to other risk and etiological factors was negative. Standard electrocardiography (ECG), continuous Holter-ECG and echocardiography (both transthoracic and transesophageal) revealed no potential cardiac embolic source. Doppler ultrasonography did not reveal atherosclerotic changes of the carotid arteries. The patient had no history of alcohol or drug abuse. Treatment with gabapentin was started because the patient experienced a diffuse burning pain in the left side of her body on the subsequent days, exacerbated by touch of clothing, showering and so on. After an additional 6 days she suddenly experienced numbness and tingling in her right big toe, but she was discharged from the hospital and told by the neurologist not to worry.

**Figure 2:**
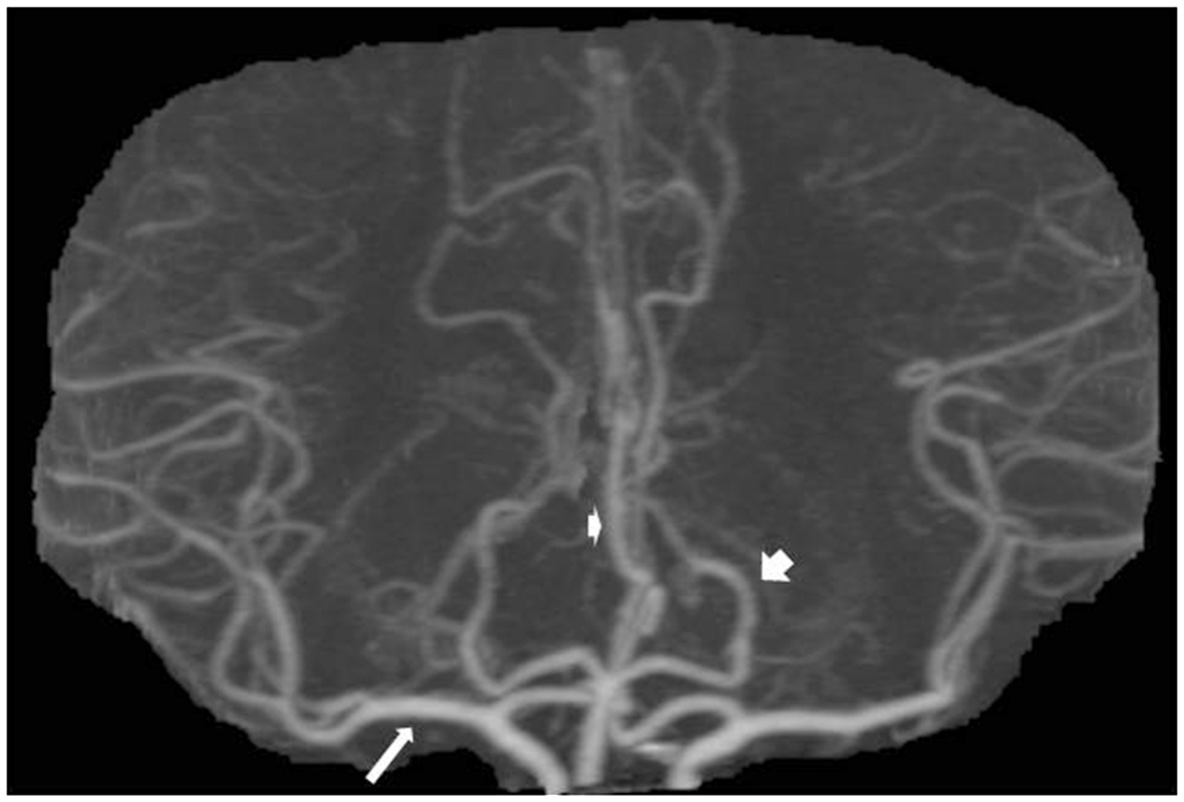
**Maximal intensity projection reconstruction of contrast-enhanced computed tomography angiography (frontal view) showing normal vessel: middle cerebral artery (arrow); anterior cerebral artery (smaller arrowhead); posterior cerebral artery (larger arrowhead). **The resolution was too low to demonstrate the vessels supplying the thalamus.

During the next few days her symptoms expanded to affect almost the whole right side of her body, including part of the face and arm. Her own doctor was perceived to be reluctant, but after several visits she was re-hospitalized. On examination this time she reported identical symptoms as before, but now bilaterally. Touch in the area below T5 on the right side was even more painful than on the left side with known allodynia. Speech, power, reflexes, and so on were still normal, but a mild ataxia was found in both legs. MRI now revealed a new infarction in the left thalamus (Figure 1b). Examinations of the intracranial vessels with transcranial Doppler sonography and computed tomography (CT) (Figure 2) were performed with negative results.

The gabapentin dose was increased, but after being discharged from the hospital the second time her pain became excruciating. Treatment was switched to pregabalin, supplied with amitriptyline, and later tramadol, but 5 months after the first stroke the patient still experienced global dysesthesia and unbearable pain. The treatment combination of buprenorphine transdermal patches (5μg/hour), amitriptyline (35mg each evening) and gabapentin (2700mg/day) was partly successful. However, on this regime she still scored 24 out of 35 on painDETECT [7].

## Discussion

The present case illustrates that pure sensory lacunar infarcts, most often located in the ventral posterior tier nuclei which is the main sensory relay nuclei to the cerebrum [1,8], should not be considered benign. Sensory symptoms that may appear as harmless may easily be ignored, but acute sensory symptoms defined with a remarkable midline split, especially when both head and trunk are involved, is often considered unique to thalamic or thalamocortical lesions [9], and augur a minor stroke. Bilateral thalamic lesions are in general uncommon [10]. However, bilateral paramedian thalamic infarcts, due to occlusion of the artery of Percheron, are well known [11], but lateral ‘mirror lesions’ as seen in the present patient must be considered very rare. Combined with a Déjerine–Roussy syndrome [6], the case is probably unique. The only case we have found in the literature was that of Cordery and Rossor (1999) who described a patient with bilateral thalamic pain secondary to bilateral thalamic infarcts, but the lesions were not well-located and not demonstrated radiologically [12].

Establishing exact stroke mechanisms is very difficult. Despite thorough investigations, only plausible mechanisms may be launched in the majority of cases. The thalamus is supplied by very small arteries susceptible to slowly evolving lipohyalinosis and microthrombosis [13]. The principal inferolateral arteries, arising from the P2 branch of the posterior cerebral artery, supply the major part of the thalamic sensory nuclei [13]. In the present case, no focal atherosclerotic posterior artery disease was seen on CT-angiography and, as expected, we were not able to identify the principal inferolateral arteries. The size of the thalamoperforating branches of the posterior cerebral arteries varies from 100 to 400μm in diameter [14], and the artery of Percheron has only a few times been successfully demonstrated on conventional angiography [11].

On the basis of the clinical history, risk profile, the absence of findings of large artery disease and potential cardiac embolic source, small vessel disease was the most plausible cause of the lacunes in the present case. One might only speculate if an occlusion due to thrombosis *in situ* of either an unpaired (anomalous) inferolateral artery or two separate arteries occurred. Diabetes seems to be a strong predictor for relatively early recurrence of a lacunar infarction in the wake of a first ever lacunar stroke [15]. One might also argue that there is increased risk of a new sensory stroke due to the fact that thalamoperforating branches of the posterior cerebral arteries in general are of smaller caliber than the lenticulostriate vessels [14], but this hypothesis has to be confirmed in future studies. However, in a follow-up study of 695 patients who had a first ever lacunar infarction [15], the mean time for recurrence was 58 months (range 2–240, n=122) [15]. No patient in that study experienced a new lacune within the first 2 months. The odds of getting bilateral, almost identical infarctions from two different and small vascular territories within 2 weeks as in the present case must thus be considered microscopic.

A good outcome after a pure sensory stroke is reported in the literature to be the rule [3,9,14], and reassurances are often given. However, the occurrence of central post-stroke pain is particularly high when the VPN and the trigeminal spinal nucleus and tract are involved (as in lateral medullary syndrome) [16]. Amitriptyline is the best documented drug for central post-stroke pain, gabapentin is an alternative, but any drug may give poor results [16]. Despite a combination of the two mentioned above and an opioid analgesic, the patient experienced severe pain. However, her pain was reduced when she was in activity, and actually buprenorphine in double dose (10μg/hour) made here tired and inactive. Due to this, she did not want to change her medical regime, but supplied the treatment with 300 to 600mg gabapentin when needed.

Combining clinical information with radiological findings, one may outline a rather precise location of the infarctions in the present case. Hypersensitivity to pinprick and thermal stimuli indicates increased activity in the spinothalamic tracts. The ventroposterolateral nuclei relay the stimuli from the trunk and extremities, and the ventroposteriomedial nuclei relay the stimuli from the head and face to the primary sensory cortex of the parietal lobe [17]. The small ataxic component and the normal position sense found provide reasons to believe that the infarctions also may have affected areas that convey cerebellar fibers to motor-related cortices [13]. Studying the MRI images, using both an atlas [18] and reference structures [19], there is no doubt that there is good correlation between clinical and radiological findings. This case thus shows that the thalamus plays a pivotal role in central pain, but whether it acts as a generator or as a defect modulator of ascending inputs is not clear. Spontaneous pain with an intact spinothalamic tract may support the former, but hypersensitivity to touch may support the latter.

## Conclusions

This case report highlights the fact that pain may occur as a result of a small lesion in the brain. As demonstrated in the literature many times before, a lacunar infarct confined to the posterolateral thalamus was the cause. However, probably unique is the demonstration of bilateral thalamic pain secondary to two almost identical thalamic lacunes.

## Consent

Written informed consent was obtained from the patient for publication of this manuscript and accompanying images. A copy of the written consent is available for review by the Editor-in Chief of this journal.

## Competing interests

The authors declare that they have no competing interests.

## Author’s contributions

KBA examined and treated the patient from her second stay in hospital. JFP interpreted the neuroimages and contributed in writing the manuscript. Both authors read and approved the final manuscript.
